# Effects of Collagen–Glycosaminoglycan Mesh on Gene Expression as Determined by Using Principal Component Analysis-Based Unsupervised Feature Extraction

**DOI:** 10.3390/polym13234117

**Published:** 2021-11-26

**Authors:** Y-h. Taguchi, Turki Turki

**Affiliations:** 1 Department of Physics, Chuo University, Tokyo 112-8551, Japan; 2 Department of Computer Science, King Abdulaziz University, Jeddah 21589, Saudi Arabia; tturki@kau.edu.sa

**Keywords:** feature extraction, tissue engineering, microarray data, applications in biology and medicine

## Abstract

The development of the medical applications for substances or materials that contact cells is important. Hence, it is necessary to elucidate how substances that surround cells affect gene expression during incubation. In the current study, we compared the gene expression profiles of cell lines that were in contact with collagen–glycosaminoglycan mesh and control cells. Principal component analysis-based unsupervised feature extraction was applied to identify genes with altered expression during incubation in the treated cell lines but not in the controls. The identified genes were enriched in various biological terms. Our method also outperformed a conventional methodology, namely, gene selection based on linear regression with time course.

## 1. Introduction

Several factors are known to affect cell division; one such effective factor is contact with solid materials (or substance) [1]. Regulating the cell division process using biomaterials is the central theme of tissue engineering. The effect of tissue engineering scaffolds is especially important because tissue engineering cannot be conducted without equipment that can store cell lines. Collagen–glycosaminoglycan mesh is one such important biomaterial because it is used to aid wound healing [2]. Although Klappericha and Bertozzi [3] once investigated the effect of collagen–glycosaminoglycan mesh on cell division cycles using microarray analysis, the small number of samples studied prevented them from identifying genes whose expression significantly varied during development and whose expression profiles were distinct between controls and treated cells. Although they selected genes associated with *p*-values of less than 0.001, considering the number of genes as $10^{4}$, it is far below significant.

The recently proposed principal component analysis (PCA)-based unsupervised feature extraction (FE) [4] has the ability to identify genes with expression profiles that are significantly different using a small number of samples. In this study, we successfully applied PCA-based unsupervised FE to determine gene expression profiles during the cell division of cells in control conditions and in contact with collagen–glycosaminoglycan mesh. The identified genes were found to be associated with several enrichment terms with considerable biological significance.

## 2. Materials and Methods

### 2.1. Gene Expression Profiles

Gene expression profiles were downloaded from the Gene Expression Omnibus (GEO) database (GEO ID: GSE6432). The dataset in GSE6432_series_matrix.txt.gz is available in the Series Matrix File(s) section. It consists of 32 gene expression profiles of the IMR90 cell lines, and the relevant details are provided in Table 1.

### 2.2. PCA-Based Unsupervised FE

Gene expression profiles are formatted as matrices $x_{ij}\in R^{22,283\times 19}$ for treated cells and $x_{ij}\in R^{22,283\times 13}$ for control cells, where $x_{ij}$ denotes the gene expression of the *i*th probe at the *j*th sample. Before applying singular value decomposition (SVD), they were standardized as

(1) $$\begin{array}{ccc} \sum_{i}x_{ij} & = & 0 \end{array}$$

(2) $$\begin{array}{ccc} \sum_{i}x_{ij}^{2} & = & 22,283 \end{array}$$

After applying SVD, we obtained the left-hand singular value vector $u_{\ell i}$, which corresponded to the principal component score attributed to the probes, and the right-hand singular value vector $v_{\ell j}$, which corresponded to the principal component loadings attributed to the samples, if we interpreted the application of SVD as PCA.

In order to see which $v_{\ell j}$ is coincident with time points, we applied linear regression as

(3) $$v_{\ell j}=a_{\ell }+b_{\ell }t_{j},$$

where $a_{\ell }$ and $b_{\ell }$ are regression coefficients and $t_{j}$ is the time point (hours in Table 1) associated with the *j*th sample. We used the lm function in R [5], and the obtained *p*-values were corrected using the Benjamini–Hochberg criterion [4]. $v_{3j}$ for treated cell is associated with the adjusted *p*-values less than 0.05, whereas $v_{\ell j}$s for control cell is not associated with adjusted *p*-values less than 0.05. This result is appropriate because the simple cell division process may not be associated with any time development other than cell senescence [6], which might not be detected in only 24 h.

Probes are selected by assuming that $u_{3i}$, associated with $v_{3j}$, obeys the Gaussian distribution (null hypothesis) by assigning *p*-values to the probes as

(4) $$\begin{array}{c} P_{i}=P_{\chi^{2}}\left[>{\left(\frac{u_{3i}}{\sigma_{3}}\right)}^{2}\right], \end{array}$$

where $P_{\chi^{2}}\left[>x\right]$ is the cumulative $\chi^{2}$ distribution, the argument is larger than *x*, and $\sigma_{3}$ is the standard deviation. Thus, 324 probes associated with the adjusted *p*-value less than 0.01 were selected for the treated cell lines.

### 2.3. Gene Selection Using Linear Regression

As an alternative method to PCA-based unsupervised FE, we utilized linear regression-based FE. Linear regression is applied to $x_{ij}$ as

(5) $$\begin{array}{c} x_{ij}=a_{i}+b_{i}t_{j}, \end{array}$$

where $a_{i}$ and $b_{i}$ are regression coefficients and $t_{j}$ is the time point (hours in Table 1) associated with the *j*th sample. Subsequently, the adjusted *p*-values that were less than 0.01 were selected. The number of probes selected for treated cell lines was 813, and no probes were selected for the control cell lines.

### 2.4. Enrichment Analysis

The IDs of the selected probes were converted to gene symbol using the ID converter in DAVID [7]. Then, the gene symbols converted from the probe IDs were uploaded to Enrichr [8].

## 3. Results

As mentioned in the Materials and Methods Section, genes associated with the 318 probes for the treated cell lines (contact with collagen–glycosaminoglycan mesh) were uploaded to Enrichr (no probes were selected for control cell lines using this method). The full list of probes, genes, and enrichment analysis is provided in the Supplementary Materials (Data S1). Several enriched biological terms were determined.

The top ranked term in the GO biological process (BP) (Table 2) is “regulation of apoptotic process”. Na et al. reported [9] that collagen–glycosaminoglycan has an anti-apoptosis effect. Thus, the fact that this term is ranked first is reasonable.

“Focal adhesion” is the top ranked term in “GO Cellular Component 2021” (Table 3) and the nineth ranked in “KEGG 2021 Human” (Table 4); moreover, Murphy et al. [10] reported that the collagen–glycosaminoglycan scaffold plays critical roles in focal adhesion.

Other than these three categories, there are some additional categories that support the suitability of our analysis. For example, “ARCHS4 Cell-lines” lists IMR90, which is the cell line used in this study, as the top ranked cell line (Table 5).

Moreover, although it is not the top ranked term, “FETAL LUNG”, from which IMR90 cell lines were derived, is ranked within the top 10 ranked terms in “ARCHS4 Tissues” (Table 6).

Although we provide only a few examples, our results suggest that our analysis was robust.

## 4. Discussion

Although we successfully applied our methodology to the dataset, one might wonder whether more conventional methods can achieve similar performance. Since this dataset was generated using archaic technology, namely, microarray, more modernized methodologies adapted to high-throughput sequencing technology (e.g., edgeR [11] or DESeq2 [12]) cannot be employed. Moreover, the archaic technologies adapted to microarray (e.g., SAM [13] and limma [14]) cannot be employed, because they can only deal with categorical classification, whereas we need to identify genes whose expressions are altered as a numerical variable (hours). Thus, we decided to employ more conventional methodology than SAM or limma, namely, gene selection using linear regression.

As described in the Materials and Methods Section, we identified 813 probes using linear regression-based FE and uploaded the gene symbols associated with the identified probes to Enrichr. When considering only the number of probes selected, it performed better than the PCA-based unsupervised FE, which could only identify 324 probes. Selecting no probes for the control cell lines is the same as PCA-based unsupervised FE. Thus, it seems that the application of PCA-based unsupervised FE, instead of linear regression, was not productive.

Nevertheless, if we consider the performance of the enrichment analysis more carefully, this impression is reversed. A full list of the probes, genes, and the results of enrichment analysis are provided in the Supplementary Materials (Data S2). First, for “GO BP 2021”, in which PCA-based unsupervised FE ranked apoptosis first (Table 2 and Table 7), although the top ranked term “regulation of apoptotic process” in Table 2 is associated with the adjusted *p*-value as small as $2.52\times 10^{-13}$, the top ranked term in Table 7 is associated with adjusted *p*-value as large as $4.56\times 10^{-2}$, which is much less significant. Even the tenth ranked term in Table 2 is more significant than the top ranked term in Table 7. Generally, more genes uploaded have more opportunities to be associated with more significant enrichment. Nevertheless, genes associated with 813 probes, which were greater than the 324 probes identified using PCA-based unsupervised FE, could be associated with the less significant terms. This clearly suggests the inferiority of linear regression as compared to PCA-based unsupervised FE.

Regarding the comparison of the “GO Cellular Component 2021” in Table 3 and Table 8, we have a similar impression.

Although “focal adhesion” is ranked first in both Tables, its significance is very distinct. It is associated with an adjusted *P*-value as small as $4.77\times 10^{-27}$ in Table 3, whereas it is associated with that as large as $3.39\times 10^{-7}$ in Table 8. The number of overlapping genes is only 39 in Table 8, whereas it is higher (43) in Table 3, despite the fact that a higher total number of genes was uploaded to Enrichr, as shown in Table 8. Thus, the performance of linear regression is again poorer than that of PCA-based unsupervised FE.

For KEGG, not only are the generally adjusted *p*-values larger (i.e., less significant) in Table 9 than those in Table 4, but also “Glycolysis/Gluconeogenesis” and “Focal adhesion”, which are ranked within the top 10 in Table 4, are not even listed in Table 9, and no other terms seemingly related to the experiments are mentioned. Thus, the performance of linear regression is again poorer than that of PCA-based unsupervised FE.

For “ARCHS4 Cell-lines” and “ARCHS4 Tissue”, the results are similar. In Table 10, not only are the adjusted *p*-values generally larger (i.e., less significant) than those in Table 5, but the adjusted *p*-values attributed to IMR90 in Table 10 ($1.06\times 10^{-5}$) are also much larger (i.e., less significant) than those in Table 5. The number of overlapping genes for IMR90 is only 128 in Table 5, whereas that in Table 10 is 89, despite the fact that more than twice the total number of genes were uploaded to Enrichr, as shown in Table 5. However, the number of overlapping genes for HUVEC, which is the wrong one, is as large as 113 in Table 10, whereas that in Table 5 is only 64. Thus, the increased number of genes selected using linear regression substantially contributes to the increase in overlapping genes assigned to the wrong answer. Moreover, lower ranked terms failed to demonstrate an association with significant *p*-values (e.g., less than 0.015). These finding suggest the inferiority of linear regression as compared to PCA-based unsupervised FE.

Although “FETAL LUNG” is fourth ranked in Table 11, its adjusted *p*-value is $1.05\times 10^{-3}$, which is much less significant than that in Table 6 ($9.58\times 10^{-9}$). Thus, overall, PCA-based unsupervised FE performed better than linear regression.

Finally, we attempted to conduct a time-series analysis, which is more widely used than linear regression for time course data. To this end, we used the fsMTS [15] package implemented in R [5] that included multiple methods, such as correlation-based, lasso-based, mutual information-based, and random forest-based methods. Nevertheless, none of the fsMTS methods could be performed. This was because time-series analysis requires auto/cross-correlations that require the memory size proportional to the square of the number of features. Since the number of features in this analysis was as high as $10^{4}$, it was computationally infeasible to execute the methods in fsMTS. Thus, our strategy, PCA-based unsupervised FE, was the only one applicable to the present data set.

The limitation of our methodology is that because of its unsupervised nature, when it fails to select biologically reasonable genes, there are no ways to improve it, although it occasionally worked effectively in the present study.

## 5. Conclusions

In the current study, we applied PCA-based unsupervised FE to gene expression profiles for IMR90 cell lines incubated in collagen–glycosaminoglycan mesh. Whereas no genes whose expressions vary over time were detected in control cell lines, the expression profiles of several genes were altered during the cell division process. These genes are associated with several enriched biological terms. One conventional method, linear regression, was employed for comparison. Although it could select several hundred genes whose expressions vary over time, their enrichment was inferior to that seen using PCA-based unsupervised FE. Thus, not only can PCA-based unsupervised FE achieve a good performance, but it can also outperform a conventional method. We demonstrated that collagen–glycosaminoglycan is an effective medium that could be used for cell culture.

## Figures and Tables

**Table 1:** The number of samples with the gene expression profiles. Treated means contact with the collagen–glycosaminoglycan mesh.

Conditions	1 h	2 h	4 h	8 h	12 h	24 h	48 h	Total
treated	3	2	3	4	3	2	2	19
control	1	2	3	2	3	1	1	13

**Table 2:** The top 10 enriched terms in “GO Biological Process 2021” using Enrichr. Overlap is the number of common genes between the genes uploaded and the genes in the category divided by the number of genes in the category. Probes, whose associated genes were uploaded to Enrichr, were identified using PCA-based unsupervised FE.

Term	Overlap	*p*-Value	Adjusted *p*-Value
regulation of apoptotic process (GO:0042981)	41/742	$1.18\times 10^{-16}$	$2.52\times 10^{-13}$
SRP-dependent cotranslational protein targeting to membrane (GO:0006614)	17/90	$4.06\times 10^{-16}$	$4.33\times 10^{-13}$
cotranslational protein targeting to membrane (GO:0006613)	17/94	$8.78\times 10^{-16}$	$6.24\times 10^{-13}$
protein targeting to ER (GO:0045047)	17/103	$4.36\times 10^{-15}$	$2.33\times 10^{-12}$
cytoplasmic translation (GO:0002181)	16/93	$1.44\times 10^{-14}$	$6.15\times 10^{-12}$
nuclear-transcribed mRNA catabolic process, nonsense-mediated decay (GO:0000184)	17/113	$2.16\times 10^{-14}$	$7.70\times 10^{-12}$
cellular protein metabolic process (GO:0044267)	27/417	$7.60\times 10^{-13}$	$2.10\times 10^{-10}$
peptide biosynthetic process (GO:0043043)	18/162	$7.89\times 10^{-13}$	$2.10\times 10^{-10}$
negative regulation of programmed cell death (GO:0043069)	25/381	$4.24\times 10^{-12}$	$1.00\times 10^{-9}$
nuclear-transcribed mRNA catabolic process (GO:0000956)	17/171	$2.11\times 10^{-11}$	$4.51\times 10^{-9}$

**Table 3:** The top 10 enriched terms in “GO Cellular Component 2021” using Enrichr. Overlap is the number of common genes between the genes uploaded and the genes in the category divided by the number of genes in the category. Probes, whose associated genes were uploaded to Enrichr, were identified using PCA-based unsupervised FE.

Term	Overlap	*p*-Value	Adjusted *p*-Value
focal adhesion (GO:0005925)	43/387	$2.22\times 10^{-29}$	$4.77\times 10^{-27}$
cell-substrate junction (GO:0030055)	43/394	$4.69\times 10^{-29}$	$5.04\times 10^{-27}$
intracellular organelle lumen (GO:0070013)	40/848	$5.63\times 10^{-14}$	$4.03\times 10^{-12}$
collagen-containing extracellular matrix (GO:0062023)	25/380	$4.00\times 10^{-12}$	$2.15\times 10^{-10}$
endoplasmic reticulum lumen (GO:0005788)	21/285	$2.78\times 10^{-11}$	$1.19\times 10^{-9}$
cytosolic large ribosomal subunit (GO:0022625)	10/55	$7.95\times 10^{-10}$	$2.85\times 10^{-8}$
large ribosomal subunit (GO:0015934)	10/59	$1.64\times 10^{-9}$	$5.03\times 10^{-8}$
ribosome (GO:0005840)	10/62	$2.72\times 10^{-9}$	$7.30\times 10^{-8}$
secretory granule lumen (GO:0034774)	19/316	$7.48\times 10^{-9}$	$1.79\times 10^{-7}$
ficolin-1-rich granule lumen (GO:1904813)	12/123	$2.50\times 10^{-8}$	$5.37\times 10^{-7}$

**Table 4:** The top 10 enriched terms in “KEGG 2021 Human” using Enrichr. Overlap is the number of common genes between the genes uploaded and the genes in the category divided by the number of genes in the category. Probes, whose associated genes were uploaded to Enrichr, were identified using PCA-based unsupervised FE.

Term	Overlap	*p*-Value	Adjusted *p*-Value
Coronavirus disease	21/232	$5.36\times 10^{-13}$	$1.22\times 10^{-10}$
Ribosome	17/158	$5.88\times 10^{-12}$	$6.67\times 10^{-10}$
Legionellosis	9/57	$2.09\times 10^{-8}$	$1.58\times 10^{-6}$
Salmonella infection	16/249	$4.75\times 10^{-8}$	$2.70\times 10^{-6}$
IL-17 signaling pathway	10/94	$1.64\times 10^{-7}$	$7.46\times 10^{-6}$
Glycolysis/Gluconeogenesis	8/67	$1.20\times 10^{-6}$	$4.52\times 10^{-5}$
Lipid and atherosclerosis	13/215	$1.75\times 10^{-6}$	$5.69\times 10^{-5}$
Protein digestion and absorption	9/103	$3.65\times 10^{-6}$	$1.04\times 10^{-4}$
Focal adhesion	12/201	$5.03\times 10^{-6}$	$1.17\times 10^{-4}$
PI3K-Akt signaling pathway	16/354	$5.14\times 10^{-6}$	$1.17\times 10^{-4}$

**Table 5:** The top 10 enriched terms in “ARCHS4 Cell-lines” using Enrichr. Overlap is the number of common genes between the genes uploaded and the genes in the category divided by the number of genes in the category. Probes, whose associated genes were uploaded to Enrichr, were identified using PCA-based unsupervised FE.

Term	Overlap	*p*-Value	Adjusted *p*-Value
IMR90	89/2395	$1.56\times 10^{-24}$	$1.95\times 10^{-22}$
NHDF	79/2395	$2.70\times 10^{-18}$	$1.69\times 10^{-16}$
BJ CELL	72/2395	$2.02\times 10^{-14}$	$8.41\times 10^{-1}$3
HUVEC	64/2395	$1.61\times 10^{-10}$	$5.04\times 10^{-9}$
T24	62/2395	$1.23\times 10^{-9}$	$3.09\times 10^{-8}$
T98G	59/2395	$2.22\times 10^{-8}$	$4.63\times 10^{-7}$
BT549	56/2395	$3.28\times 10^{-7}$	$5.12\times 10^{-6}$
DU145	56/2395	$3.28\times 10^{-7}$	$5.12\times 10^{-6}$
CAKI1	55/2395	$7.68\times 10^{-7}$	$9.60\times 10^{-6}$
U87	55/2395	$7.68\times 10^{-7}$	$9.60\times 10^{-6}$

**Table 6:** The top 10 enriched terms in “ARCHS4 Tissues” using Enrichr. Overlap is the number of common genes between the genes uploaded and the genes in the category divided by the number of genes in the category. Probes, whose associated genes were uploaded to Enrichr, were identified using PCA-based unsupervised FE.

Term	Overlap	*p*-Value	Adjusted *p*-Value
MYOBLAST	87/2316	$3.16\times 10^{-24}$	$3.38\times 10^{-22}$
FIBROBLAST	86/2316	$1.45\times 10^{-23}$	$7.76\times 10^{-22}$
FORESKIN FIBROBLAST	72/2316	$3.58\times 10^{-15}$	$1.28\times 10^{-13}$
BLOOD DENDRITIC CELLS	66/2316	$4.31\times 10^{-12}$	$1.15\times 10^{-10}$
DENDRITIC CELL	63/2316	$1.13\times 10^{-10}$	$2.02\times 10^{-9}$
OSTEOBLAST	63/2316	$1.13\times 10^{-10}$	$2.02\times 10^{-9}$
KUPFFER CELL	62/2316	$3.22\times 10^{-10}$	$4.30\times 10^{-9}$
VASCULAR SMOOTH MUSCLE	62/2316	$3.22\times 10^{-10}$	$4.30\times 10^{-9}$
FETAL LUNG	61/2316	$8.95\times 10^{-10}$	$9.58\times 10^{-9}$
LIVER (BULK TISSUE)	61/2316	$8.95\times 10^{-10}$	$9.58\times 10^{-9}$

**Table 7:** The top 10 enriched terms in “GO Biological Process 2021” using Enrichr. Overlap is the number of common genes between the genes uploaded and the genes in the category divided by the number of genes in the category. Probes, whose associated genes were uploaded to Enrichr, were identified using linear regression.

Term	Overlap	*p*-Value	Adjusted *p*-Value
actin polymerization or depolymerization (GO:0008154)	9/50	$4.05\times 10^{-5}$	$4.56\times 10^{-2}$
rRNA-containing ribonucleoprotein complex export from nucleus (GO:0071428)	4/7	$4.22\times 10^{-5}$	$4.56\times 10^{-2}$
positive regulation of protein modification process (GO:0031401)	20/214	$4.32\times 10^{-5}$	$4.56\times 10^{-2}$
transmembrane receptor protein tyrosine kinase signaling pathway (GO:0007169)	30/404	$5.44\times 10^{-5}$	$4.56\times 10^{-2}$
protein stabilization (GO:0050821)	17/179	$1.31\times 10^{-4}$	$7.08\times 10^{-2}$
regulation of lipid biosynthetic process (GO:0046890)	7/35	$1.46\times 10^{-4}$	$7.08\times 10^{-2}$
regulation of cellular metabolic process (GO:0031323)	8/47	$1.63\times 10^{-4}$	$7.08\times 10^{-2}$
positive regulation of cellular protein metabolic process (GO:0032270)	12/102	$1.74\times 10^{-4}$	$7.08\times 10^{-2}$
regulation of stress fiber assembly (GO:0051492)	10/74	$1.90\times 10^{-4}$	$7.08\times 10^{-2}$
regulation of mRNA catabolic process (GO:0061013)	13/122	$2.58\times 10^{-4}$	$7.65\times 10^{-2}$

**Table 8:** The top 10 enriched terms in “GO Cellular Component 2021” using Enrichr. Overlap is the number of common genes between the genes uploaded and the genes in the category divided by the number of genes in the category. Probes, whose associated genes were uploaded to Enrichr, were identified using linear regression.

Term	Overlap	*p*-Value	Adjusted *p*-Value
focal adhesion (GO:0005925)	39/387	$1.41\times 10^{-9}$	$3.39\times 10^{-7}$
cell-substrate junction (GO:0030055)	39/394	$2.34\times 10^{-9}$	$3.39\times 10^{-7}$
actin cytoskeleton (GO:0015629)	25/316	$8.27\times 10^{-5}$	$7.97\times 10^{-3}$
intracellular organelle lumen (GO:0070013)	49/848	$2.02\times 10^{-4}$	$1.46\times 10^{-2}$
nucleus (GO:0005634)	186/4484	$1.06\times 10^{-3}$	$5.51\times 10^{-2}$
collagen-containing extracellular matrix (GO:0062023)	25/380	$1.30\times 10^{-3}$	$5.51\times 10^{-2}$
cytoplasmic stress granule (GO:0010494)	8/65	$1.54\times 10^{-3}$	$5.51\times 10^{-2}$
intracellular membrane-bounded organelle (GO:0043231)	210/5192	$1.66\times 10^{-3}$	$5.51\times 10^{-2}$
endoplasmic reticulum lumen (GO:0005788)	20/285	$1.81\times 10^{-3}$	$5.51\times 10^{-2}$
intracellular non-membrane-bounded organelle (GO:0043232)	58/1158	$1.91\times 10^{-3}$	$5.51\times 10^{-2}$

**Table 9:** The top 10 enriched terms in “KEGG 2021 Human” using Enrichr. Overlap is the number of common genes between the genes uploaded and the genes in the category divided by the number of genes in the category. Probes, whose associated genes were uploaded to Enrichr, were identified using linear regression.

Term	Overlap	*p*-Value	Adjusted *p*-Value
PI3K-Akt signaling pathway	28/354	$3.17\times 10^{-5}$	$9.30\times 10^{-3}$
Sphingolipid signaling pathway	13/119	$2.01\times 10^{-4}$	$1.66\times 10^{-2}$
Arrhythmogenic right ventricular cardiomyopathy	10/77	$2.65\times 10^{-4}$	$1.66\times 10^{-2}$
Antigen processing and presentation	10/78	$2.95\times 10^{-4}$	$1.66\times 10^{-2}$
Hepatitis C	15/157	$2.99\times 10^{-4}$	$1.66\times 10^{-2}$
Salmonella infection	20/249	$3.39\times 10^{-4}$	$1.66\times 10^{-2}$
Hippo signaling pathway	15/163	$4.47\times 10^{-4}$	$1.87\times 10^{-2}$
Tight junction	15/169	$6.54\times 10^{-4}$	$2.29\times 10^{-2}$
Protein processing in endoplasmic reticulum	15/171	$7.39\times 10^{-4}$	$2.29\times 10^{-2}$
AMPK signaling pathway	12/120	$7.81\times 10^{-4}$	$2.29\times 10^{-2}$

**Table 10:** The top 10 enriched terms in “ARCHS4 Cell-lines” using Enrichr. Overlap is the number of common genes between the genes uploaded and the genes in the category divided by the number of genes in the category. Probes, whose associated genes were uploaded to Enrichr, were identified using linear regression.

Term	Overlap	*p*-Value	Adjusted *p*-Value
IMR90	128/2395	$8.49\times 10^{-8}$	$1.06\times 10^{-5}$
HUVEC	113/2395	$1.54\times 10^{-4}$	$9.65\times 10^{-3}$
NHDF	112/2395	$2.35\times 10^{-4}$	$9.78\times 10^{-3}$
BT549	103/2395	$6.32\times 10^{-3}$	$1.62\times 10^{-1}$
BJ CELL	101/2395	$1.17\times 10^{-2}$	$1.62\times 10^{-1}$
HNSCC	101/2395	$1.17\times 10^{-2}$	$1.62\times 10^{-1}$
KNS42	101/2395	$1.17\times 10^{-2}$	$1.62\times 10^{-1}$
NHBE	101/2395	$1.17\times 10^{-2}$	$1.62\times 10^{-1}$
U87	101/2395	$1.17\times 10^{-2}$	$1.62\times 10^{-1}$
DAOY	99/2395	$2.07\times 10^{-2}$	$2.35\times 10^{-1}$

**Table 11:** The top 10 enriched terms in “ARCHS4 Tissues” using Enrichr. Overlap is the number of common genes between the genes uploaded and the genes in the category divided by the number of genes in the category. Probes, whose associated genes were uploaded to Enrichr, were identified using linear regression.

Term	Overlap	*p*-Value	Adjusted *p*-Value
FIBROBLAST	116/2316	$9.27\times 10^{-6}$	$5.01\times 10^{-4}$
VENTRICLE	116/2316	$9.27\times 10^{-6}$	$5.01\times 10^{-4}$
ADIPOSE (BULK TISSUE)	113/2316	$3.89\times 10^{-5}$	$1.05\times 10^{-3}$
FETAL LUNG	113/2316	$3.89\times 10^{-5}$	$1.05\times 10^{-3}$
RESPIRATORY SMOOTH MUSCLE	112/2316	$6.14\times 10^{-5}$	$1.33\times 10^{-3}$
OMENTUM	111/2316	$9.58\times 10^{-5}$	$1.73\times 10^{-3}$
SUBCUTANEOUS ADIPOSE TISSUE	106/2316	$7.60\times 10^{-4}$	$1.17\times 10^{-2}$
MYOBLAST	104/2316	$1.61\times 10^{-3}$	$2.18\times 10^{-2}$
NEURONAL EPITHELIUM	103/2316	$2.31\times 10^{-3}$	$2.77\times 10^{-2}$
ASTROCYTE	99/2316	$8.75\times 10^{-3}$	$8.54\times 10^{-2}$

## Data Availability

Data used in this study are available in GEO ID GSE6432.

## Supplementary Materials

The following are available at https://www.mdpi.com/article/10.3390/polym13234117/s1, Data S1: The full list of probes, genes, and enrichment analysis is provided in the supplementary material for PCA based unsupervised FE, Data S2: The full list of probes, genes, and enrichment analysis is provided in the supplementary material for linear regression.
